# Pediatric primary intraventricular hemorrhage: A case report of isolated fourth ventricle hemorrhage in a 10‐year‐old boy

**DOI:** 10.1002/ccr3.7952

**Published:** 2023-09-25

**Authors:** Aswith Das, Biju Bhadran, Vivek Sanker, Vinay Suresh, Pratik Agarwal, Tirth Dave

**Affiliations:** ^1^ MCh Neurosurgery Government TD Medical College Hospital Alappuzha India; ^2^ Team Erevnites Trivandrum India; ^3^ Department of Neurosurgery Government Medical College Trivandrum India; ^4^ Noorul Islam Institute of Medical Sciences Trivandrum India; ^5^ King George's Medical University Lucknow India; ^6^ Lokmanya Tilak Municipal Medical College and General Hospital Mumbai India; ^7^ Bukovinian State Medical University Chernivtsi Ukraine

**Keywords:** external ventricular drain, pediatric intraventricular hemorrhage, pediatric neurosurgery, ventriculoperitoneal shunt

## Abstract

**Key Clinical Message:**

Primary intraventricular hemorrhage (PIVH) is a rare condition in pediatric patients, presenting with headache, vomiting, and altered mental status. Surgical interventions, such as external ventricular drain placement, followed by ventriculoperitoneal shunting, show promising outcomes. Further research is needed to enhance understanding and optimize management strategies for pediatric PIVH.

**Abstract:**

This case report describes a 10‐year‐old boy with isolated primary intraventricular hemorrhage (PIVH) in the fourth ventricle, shedding light on its clinical presentation and management challenges. The patient presented with headache, vomiting, and altered sensorium, and was subsequently diagnosed with obstructive hydrocephalus due to intraventricular bleeding. Emergency external ventricular drain (EVD) insertion was performed, followed by ventriculoperitoneal shunt placement, resulting in a favorable outcome. The etiology of PIVH in children differs from that in adults, with arteriovenous malformations, Moyamoya disease, and aneurysms being commonly implicated causes. Management strategies for pediatric PIVH are challenging due to limited research, but EVD placement and surgical interventions have shown promise.

## Graphical abstract

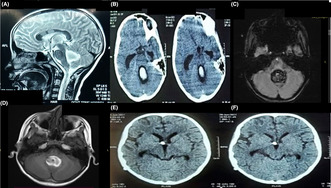

## 1 INTRODUCTION

Primary intraventricular hemorrhage (PIVH) refers to bleeding that occurs specifically within the ventricular system of the brain, without any involvement or bleeding within the surrounding brain tissue. It is a rare occurrence comprising approximately 3.1% of nontraumatic central nervous system hemorrhages.^1^ While there is some literature on the etiology, clinical features, and management of PIVH in adults, the understanding of this condition in the pediatric population (Table 1) is limited, posing challenges in identifying its characteristics and developing specific management protocols. Available reports suggest that arteriovenous malformations (AVMs), Moyamoya disease, and aneurysms are among the commonly implicated causes in pediatric cases.^2^

**TABLE 1:** Etiological factors of adult and pediatric intraventricular hemorrhage.

Etiological factors	IVH in adults	IVH in children
Underlying cause	IVH usually occurs as a secondary phenomenon when parenchymal or ICH ruptures into the ventricular space or when SAH extends into the ventricles.	Intracranial vascular malformations account for 40%–90% of Pediatric ICH (pICH) etiology and are dominated by AVMs^3^, ^4^
Vascular malformations	Vascular malformations are the most frequently identified cause of primary IVH in adults^5^, ^6^, ^7^, ^8^, ^9^, ^10^, ^11^, ^12^, ^13^, ^14^, ^15^	Arteriovenous malformation^2^
Intraventricular tumors	Intraventricular tumors (papilloma, neurocytoma, meningioma, metastases, astrocytoma, ependymoma) have been reported as causes of primary IVH in adults^8^, ^16^, ^17^, ^18^, ^19^, ^20^, ^21^, ^22^	The incidence of brain neoplasm‐related ICH is known to be low in adults (<5%), and likely lower in children
Intraventricular aneurysms	Intraventricular aneurysms developing within the distal lenticulostriate or choroidal arteries have been reported as causes of primary IVH in adults^7^, ^18^, ^23^	Aneurysm^2^
Moyamoya disease	Moyamoya disease has been reported as a cause of primary IVH in adults^5^, ^6^, ^18^	Moyamoya disease^2^
Coagulopathies	Coagulopathies, acquired or inherited, have been reported as causes of primary IVH in adults^6^, ^7^, ^9^, ^24^, ^25^, ^26^, ^27^	The cause also varies by age at presentation, with an overrepresentation of hemostatic disorders in the first year of life, and an increase in the relative prevalence of vascular malformations in teenagers^3^, ^28^
Pituitary apoplexy	Pituitary apoplexy has been reported as a cause of primary IVH in adults^29^	No sufficient information present
Vasculitis	Vasculitis has been reported as a cause of primary IVH in adults^30^	Primary CNS vasculitis and SLE vasculitis have been rarely associated with pICH^31^, ^32^
Fibromuscular dysplasia	Fibromuscular dysplasia has been reported as a cause of primary IVH in adults^7^	Rarely childhood SAH can be associated with fibromuscular dysplasia^33^
Sympathomimetic agents	Sympathomimetic agents have been reported as a cause of primary IVH in adults^34^, ^35^	No sufficient information present
Idiopathic	In approximately 20%–50% of cases in adults, no cause is identified^8^, ^9^, ^25^	There is a high number of patients with unknown cause of intracranial hemorrhage, exceeding 50% or 30% of reported patients^36^, ^37^, ^38^

Abbreviations: AVMs, arteriovenous malformations; ICH, intracerebral hemorrhage; IVH, intraventricular hemorrhage; SAH, subarachnoid hemorrhage.

Surgery, particularly the use of an external ventricular drain (EVD) to alleviate intracranial pressure in case of acute hydrocephalus, remains the primary approach documented in the literature.^2^ Due to the significant risks of mortality and neurological impairment associated with Primary pediatric IVH (PIVH), it is imperative to thoroughly report and discuss the key features of this condition in the pediatric population. In this context, we present a rare case of isolated PIVH in the fourth ventricle observed in a 10‐year‐old boy.

## 2 CASE REPORT

A 10‐year‐old child presented with a 1‐day history of headache and vomiting, followed by altered sensorium. He was not taking any medications and had no history of trauma. There was no history of perinatal complications, all vaccinations were administered on schedule, with timely achievement of developmental milestones. Upon initial examination, the patient did not exhibit fever, rash, or meningeal symptoms. He was hemodynamically stable, had a clear airway, and was breathing normally; his Glasgow Coma Scale was E3V2M3, both pupils were equally reactive to light with a size of 4 mm, and no papilledema or neck stiffness. However, following admission, the patient's Glasgow Coma Scale score deteriorated to E1V1M3 with pupils reacting to light bilaterally equally, necessitating intubation and elective ventilation.

**FIGURE 1:**
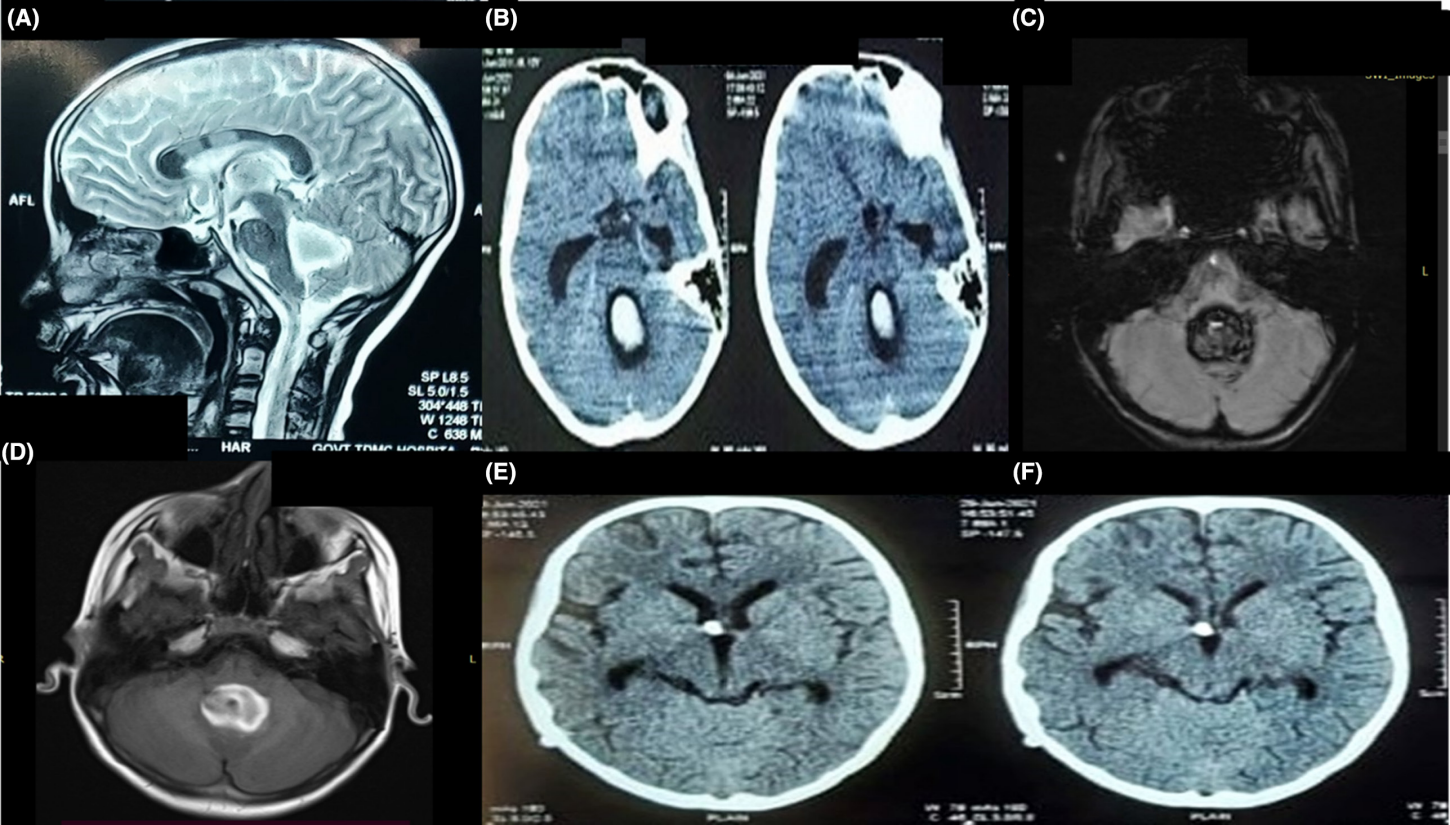
(A) Magnetic resonance imaging (MRI) showing isolated hemorrhage in the fourth ventricle. (B) CT scan showing a hyperdense area of blood density noted within the fourth ventricle, likely intraventricular hemorrhage. Upstream dilatation of bilateral lateral and third ventricle is noted, possibly due to the obstructive hydrocephalus. (C) Susceptibility weighted image showing blooming foci in the fourth ventricle. (D) T1‐weighted image showing hyperintense area within the fourth ventricle. (E, F) CT scan taken after ventriculoperitoneal shunt showing EVD tube in situ with tip in the right lateral ventricle. Mild hydrocephalus (reduced) seen.

The results of various medical tests, including complete blood count, measurements of urea and electrolyte measures, C‐reactive protein levels, glucose levels, liver function tests, and clotting profiles, were within normal ranges (Hb: 12.6 g/dL; TC: 8500; platelet count: 3.5 L; CRP: 0.6 mg/dL; PT/INR: 13.4/0.99). Initial brain scan using computed tomography (CT) revealed a recent bleeding episode in the fourth ventricle with obstructive hydrocephalus. No bleeding was observed within the brain tissue itself or in the subarachnoid space. The magnetic resonance imaging (MRI) brain confirmed the findings and magnetic resonance angiography (MRA) revealed no vascular abnormality (Figure 1A–F).

**FIGURE 2:**
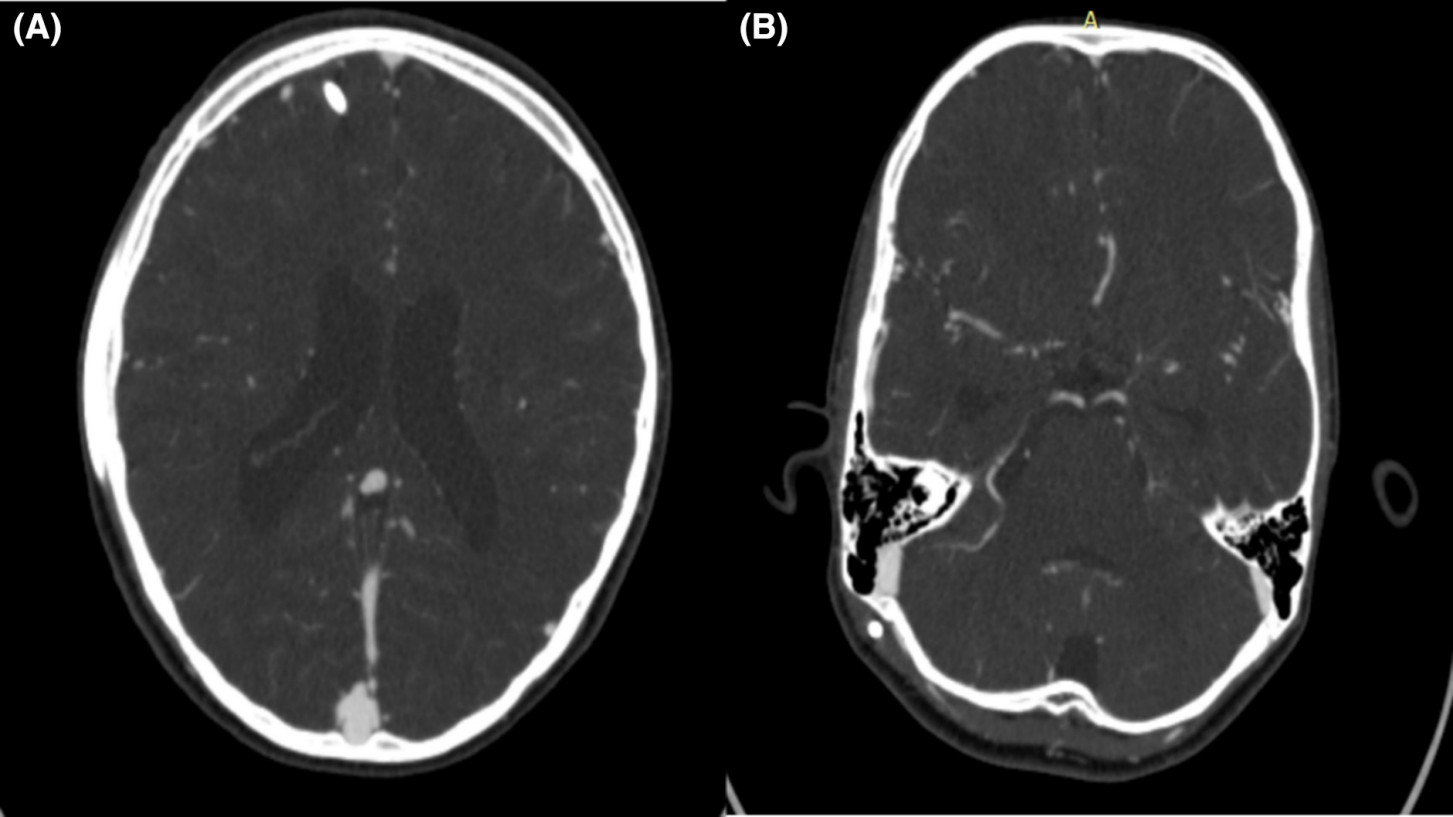
(A, B) CT angiogram taken after resolution of bleed 2 months after discharge.

Emergency EVD insertion was performed following which his GCS improved to E3VtM5 (child was intubated and connected to pressure support mode of mechanical ventilator at that time). After 6 h, he was extubated and the next day his GCS was E4V5M6. On Day 6, since there was residual hematoma in fourth ventricle in CT scan and high CSF output in EVD, his EVD was changed to ventriculoperitoneal shunt. The procedure was done under general anesthesia with propofol as induction agent. The postprocedure period was uneventful, and a repeat CT scan done showed resolving hematoma inside the fourth ventricle. At the time of discharge, the patient was symptomatically better, neurologic status improved and GCS E4V5M6, without any neurologic deficit to the patient. Post discharge, the child was closely monitored and was asymptomatic. A repeat CT taken after 60 days showed complete resolution of hematoma and a CT brain with angiogram done after 2 months (Figure 2A,B) ruled out any vascular anomalies.

## 3 DISCUSSION

PIVH is an uncommon condition characterized by bleeding within the ventricles of the brain, lacking any parenchymal or subarachnoid bleeding. While there is a growing body of research on PIVH in adults, the understanding of this condition in the pediatric population remains limited. This case report presents a 10‐year‐old child with PIVH, shedding light on the clinical presentation and management challenges.

This scarcity of studies have made it difficult to consistently identify the characteristic features of PIVH in children. Headache has consistently been reported as the most frequent presenting symptom in patients, followed by vomiting and loss of consciousness.^2^, ^39^ In contrast, a study by Weinstein et al. found altered mental status to be the most commonly reported presenting symptom, followed by headache and nausea.^1^ These findings align with our case, where the child initially presented with headache and vomiting, and altered sensorium. In adult populations, hypertension has been identified as the most common etiology for PIVH.^1^, ^40^ However, the etiology of PIVH in children differs from that in adults. Vascular malformations, such as AVMs, Moyamoya disease, and aneurysms, can lead to intraventricular hemorrhage in children.^2^ Other potential etiologic factors include coagulopathies, choroid plexus tumors and cysts, and arteritis. It is crucial to consider these underlying causes when evaluating pediatric patients with PIVH.

Management strategies for PIVH in pediatric patients pose significant challenges, as there is limited research specific to this population. In the case presented, an EVD was inserted to alleviate obstructive hydrocephalus. EVD is a commonly employed method for draining cerebrospinal fluid and reducing intracranial pressure in PIVH cases. However, it is worth noting that EVD alone may be insufficient, as blood clots often occlude the drain.^41^ In a study by Guo et al., surgical intervention was the predominant treatment modality, with 83.3% of patients undergoing surgical procedures, while the remaining received conservative treatment.^2^ The management of PIVH with obstructive hydrocephalus involves the placement of an EVD and is the preferred method of treatment.^2^ In cases involving AVMs, various surgical interventions, such as resection, endovascular embolization, or stereotactic radiation, were employed.^2^ PPIVH patients diagnosed with aneurysms underwent a combined treatment approach involving the placement of an EVD and subsequent endovascular embolization.^2^ Notably, ventriculoperitoneal shunting was not employed as part of the management strategy for any of the pediatric patients in this study.^2^

Certain prognostic factors play a significant role in determining the outcome of PIVH. In particular, the initial level of consciousness, presence of early hydrocephalus, and timely diagnosis of intracranial aneurysms can adversely affect patient outcomes. These factors may lead to severe neurological deficits and increased mortality rates. Further research is needed to identify additional prognostic indicators specific to pediatric PIVH cases, allowing for improved risk stratification and tailored management approaches.

While the presented case report highlights the successful management and favorable outcome in the reported patient, it is important to acknowledge the limitations of this study. One significant limitation is the limited comparative analysis, which is also due to the limited availability of literature on PPIVH. This prevents a comprehensive understanding of the condition and its management options. Future studies should aim to address this gap by conducting larger scale investigations and drawing comparisons with similar cases in the literature.

## 4 CONCLUSION

PIVH in pediatric patients remains a challenging condition due to its rarity and limited research on this specific population. This case report adds to the existing knowledge on pediatric PIVH by providing insights into the clinical presentation, management approach, and favorable outcome in a pediatric patient, highlighting the potential efficacy of a combined strategy involving EVD placement and subsequent ventriculoperitoneal shunting. However, further research is warranted to better understand the etiology, prognostic factors, and optimal management strategies for pediatric PIVH, enabling improved outcomes for the affected children.

## AUTHOR CONTRIBUTIONS

**Aswith Das:** Conceptualization; data curation; formal analysis; investigation; methodology; writing – original draft; writing – review and editing. **Biju Bhadran:** Conceptualization; data curation; formal analysis; investigation; methodology; project administration; writing – original draft; writing – review and editing. **Vivek Sanker:** Formal analysis; investigation; methodology; project administration; supervision; validation; visualization; writing – original draft; writing – review and editing. **Vinay Suresh:** Software; supervision; validation; visualization; writing – original draft; writing – review and editing. **Pratik Agarwal:** Methodology; project administration; supervision; validation; visualization; writing – original draft; writing – review and editing. **Tirth Dave:** Software; supervision; validation; visualization; writing – original draft; writing – review and editing.

## Funding information

None.

## CONFLICT OF INTEREST STATEMENT

The authors declare no conflicts of interest.

## ETHICS STATEMENT

Ethical approval was not required for the case report as per the country's guidelines.

## CONSENT

Written informed consent was obtained from the patient to publish this report.

## Data Availability

The data that support the findings of this study are available on request from the corresponding author. The data are not publicly available due to privacy or ethical restrictions.
